# Culture-confirmed neonatal bloodstream infections and meningitis in South Africa, 2014–19: a cross-sectional study

**DOI:** 10.1016/S2214-109X(22)00246-7

**Published:** 2022-07-12

**Authors:** Rudzani C Mashau, Susan T Meiring, Angela Dramowski, Rindidzani E Magobo, Vanessa C Quan, Olga Perovic, Anne von Gottberg, Cheryl Cohen, Sithembiso Velaphi, Erika van Schalkwyk, Nelesh P Govender, Baby GERMS-SA

**Affiliations:** a Centre for Healthcare-Associated Infections, Antimicrobial Resistance and Mycoses, National Institute for Communicable Diseases, National Health Laboratory Service, Johannesburg, South Africa; b Division of Public Health Surveillance and Response, National Institute for Communicable Diseases, National Health Laboratory Service, Johannesburg, South Africa; c Centre for Respiratory Diseases and Meningitis, National Institute for Communicable Diseases, National Health Laboratory Service, Johannesburg, South Africa; d School of Public Health, Faculty of Health Sciences, University of the Witwatersrand, Johannesburg, South Africa; e School of Pathology, Faculty of Health Sciences, University of the Witwatersrand, Johannesburg, South Africa; f Department of Paediatrics and Child Health, School of Clinical Medicine, Faculty of Health Sciences, University of the Witwatersrand, Johannesburg, South Africa; g Chris Hani Baragwanath Academic Hospital, Johannesburg, South Africa; h Department of Paediatrics and Child Health, Faculty of Medicine and Health Sciences, Stellenbosch University and Tygerberg Hospital, Cape Town, South Africa; i Division of Medical Microbiology, Faculty of Health Sciences, University of Cape Town, Cape Town, South Africa; j Institute of Infection and Immunity, St George's University of London, London, UK; k MRC Centre for Medical Mycology, University of Exeter, Exeter, UK

## Abstract

**Background:**

Few population-level estimates of invasive neonatal infections have been reported from sub-Saharan Africa. We estimated the national incidence risk, aetiology, and pathogen antimicrobial susceptibility for culture-confirmed neonatal bloodstream infections and meningitis in South Africa.

**Methods:**

We conducted a cross-sectional study of neonates (<28 days of life) admitted to neonatal or paediatric wards of 256 public sector health facilities in South Africa during 2014–19. Diagnostic pathology records from Jan 1, 2014, to Dec 31, 2019, were extracted from a national pathology data warehouse. A case was defined as a neonate with at least one positive blood or cerebrospinal fluid culture during a 14-day period. Incidence risk was calculated using annual numbers of registered livebirths. Among the causative pathogens identified, we calculated the proportion of cases attributed to each of them, as well as the rates of antibiotic susceptibility of Gram-positive and Gram-negative bacteria.

**Findings:**

Among 43 438 records of positive cultures, there were 37 631 incident cases of neonatal infection with at least one pathogen isolated. The overall incidence risk of culture-confirmed infections was 6·0 per 1000 livebirths (95% CI 6·0–6·1). The incidence risk of late-onset sepsis (days 3–27 of life) was 4·9 per 1000 livebirths (4·9–5·0) and that of early-onset sepsis (days 0–2 of life) was 1·1 per 1000 livebirths (1·1–1·1); risk ratio 4·4 (95% CI 4·3–4·5). The cause of infection differed by syndrome, timing of infection onset, facility, and province, although *Klebsiella pneumoniae* (26%), *Acinetobacter baumannii* (13%), and *Staphylococcus aureus* (12%) were the dominant pathogens overall. Gram-negative bacteria had declining susceptibility to most antibiotics over the study period.

**Interpretation:**

We found a high incidence risk of late-onset sepsis with provincial variations, predominance of *K pneumoniae*, and declining antibiotic susceptibility among Gram-negative bacteria. This national surveillance in an upper-middle-income country provides a baseline burden of neonatal infections against which the impact of future clinical and public health interventions can be measured.

**Funding:**

Bill & Melinda Gates Foundation.

## Introduction

Despite global progress in reducing deaths of children younger than 5 years from 12·7 million in 1990 to 5·2 million in 2019, neonatal mortality rates remain high.^1^ Neonates accounted for 47% of all deaths in this age group, with an estimated 6700 newborn babies dying every day in 2019.^1^ The majority of these deaths occurred in sub-Saharan Africa (42%), most often caused by preterm birth complications, followed by intrapartum-related complications and neonatal infections.^1^, ^2^ Between 1990 and 2019, sub-Saharan Africa was the only region globally that failed to reduce neonatal mortality rates, making the Sustainable Development Goal survival target of fewer than 12 deaths per 1000 livebirths unlikely to be achieved by 2030.^1^ South Africa reported a slow decline in the neonatal mortality rate from 20 per 1000 livebirths in 1990 to 11 per 1000 livebirths in 2019.^1^

Globally, almost half of pathogens causing invasive neonatal infections are resistant to the WHO-recommended first-line and second-line regimens such as penicillin plus gentamicin, and third-generation cephalosporins.^3^ This high level of antimicrobial resistance amplifies the already-high mortality associated with discordant initial antibiotic therapy and an increasing requirement for use of older antibiotics such as colistin. A challenge with using new antibiotics or reintroducing older agents is that most have not been approved for use in neonates, and thus drug dosing and scheduling are difficult.^4^ The increasing frequency of antibiotic resistance is of great concern for sub-Saharan African countries faced with insufficient access to antibiotics, a high burden of infectious diseases, inadequate resources, and weak health-care systems.^5^

Population-level estimates of the burden of neonatal infections have been reported from high-income countries but reports from low-income and middle-income countries are scarce.^6^ Data from sub-Saharan Africa on the incidence, causative pathogens, and antimicrobial susceptibility of culture-confirmed invasive neonatal infections are confined to tertiary-level institutions, and most report data on health-care-associated infections or single pathogens.^7^, ^8^ These studies are not representative of the broader hospitalised neonatal population because neonates admitted to tertiary-level institutions can differ substantially from those admitted to secondary or district-level hospitals. Reporting of population-level estimates in sub-Saharan Africa is limited by well described factors such as a paucity of denominator data (eg, patient-bed days) to calculate incidence risk, a lack of surveillance or registries for reporting neonatal infections, limited access to diagnostic microbiology laboratories in some countries, and suboptimal collection of blood and cerebrospinal fluid (CSF) specimens for culture.

**Research in context**

**Evidence before this study**

We searched Google Scholar and PubMed for research papers that were published from Jan 1, 2000, to July 31, 2021, using search terms “neonate” OR “newborn” AND “sepsis” OR “infection” OR “HAI” OR “Healthcare-associated infection” OR “bloodstream infection” OR “bacteremia” OR “meningitis”. Few studies from high-income countries and middle-income countries reported on the population-level estimates of neonatal infections. However, we did not find any population-level estimates on the burden and aetiologies of neonatal infections in sub-Saharan Africa. Most reports from sub-Saharan African countries are confined to tertiary-level institutions and many report data on health-care-associated infections or single pathogens. Understanding the burden and aetiologies of neonatal infections in sub-Saharan Africa is essential to guide future public health interventions and health resource allocation.

**Added value of this study**

To our knowledge, this is the first nationally representative analysis of the burden and causative pathogens of neonatal infection in sub-Saharan Africa. The study used population-level diagnostic pathology records from a national surveillance data warehouse of hospitalised neonates (<28 days of life) in South Africa. The results show that the national incidence risk of neonatal bloodstream infections and meningitis increased from 4·4 to 7·1 per 1000 livebirths between 2014 and 2019, with some provincial variations. The dominant pathogens were *Klebsiella pneumoniae*, *Acinetobacter baumannii*, and *Staphylococcus aureus*, and the proportions varied by infection syndrome, timing of onset of infection (early-onset *vs* late-onset), and hospital tier. The study highlights the declining susceptibility of Gram-negative bacteria to most antibiotics, with substantially reduced susceptibility to carbapenems among *A baumannii* isolates. Furthermore, the study shows a decreased susceptibility of bacterial pathogens causing early-onset and late-onset infections to the WHO-recommended first-line empirical and second-line regimens, respectively.

**Implications of all the available evidence**

The incidence risk of neonatal infections in the South African public health sector is very high and possibly even higher than reported if the observed contamination rates have masked true cases of bloodstream infections and meningitis. The decreasing susceptibility to commonly used antibiotics highlights the urgent need for research into novel antimicrobials and vaccines for neonatal infections and policies to guide the appropriate use of existing antibiotics. These data provide a baseline from which to assess future public health interventions and can guide health resource allocation at all tiers of hospital care. Weighted-incidence syndromic combination antibiogram analytical modelling of the appropriateness of the WHO first-line and second-line regimens using this national dataset should be conducted to inform guidelines.

In order to guide future public health interventions and health resource allocation for hospitalised neonates, we undertook an analysis of electronic pathology records to estimate the national incidence risk and aetiology of culture-confirmed bloodstream infections (BSI) and meningitis, and the antimicrobial susceptibility of causative pathogens, among neonates in South African public sector health-care facilities over a 6-year period.

## Methods

### Study design and participants

We conducted a cross-sectional analysis of diagnostic pathology records extracted from a national surveillance data warehouse from Jan 1, 2014, to Dec 31, 2019. The National Health Laboratory Service (NHLS) comprises a network of pathology laboratories that process clinical specimens from public sector health-care facilities across South Africa, serving an estimated 80% of the country's population. The extracted dataset included pathology records from 256 health-care facilities, where neonates were treated either in a general inpatient paediatric service or in a dedicated neonatal unit, in all nine South African provinces (appendix pp 3–4).

We included neonates aged 0–27 days if their date of birth or postnatal age was recorded. Among babies with no recorded date of birth, we only included those who were recorded to have been admitted to a hospital nursery, kangaroo mother care unit, or a neonatal intensive or high care unit. We made the assumption that all neonates who had blood or CSF specimens submitted for culture had a suspected invasive infection.

Ethics clearance was obtained from the Human Research Ethics Committee of the University of the Witwatersrand (M190320). The study protocol has been published elsewhere.^9^

### Definitions

We defined a case of culture-confirmed BSI or meningitis as illness in a neonate with a positive culture of pathogenic bacteria or fungi from a blood or CSF specimen. Cultured isolates were classified as pathogens or contaminants using the US Centers for Disease Control and Prevention (CDC) list.^10^ Following the CDC recommendations, coagulase-negative *Staphylococcus* was considered pathogenic when two or more separate cultures were isolated within 2 days of the original culture.^11^ When only one or neither of two separate cultures of coagulase-negative *Staphylococcus* were identified to species level at the diagnostic laboratory, we assumed that these organisms were the same. Early-onset sepsis (EOS) was defined as occurring within the first 3 days of life (ie, 0–2 days of life) and late-onset sepsis (LOS) was defined as occurring after day 3 of life (ie, 3–27 days of life).^12^ Multidrug-resistant bacterial isolates were defined as non-susceptible to one or more agents in at least three classes tested.

### Procedures

Among neonates with clinical signs and symptoms of an infection, at the discretion of the attending clinician, blood culture or CSF specimens (or both) were aseptically collected according to standard NHLS operating procedures.^13^ All blood and CSF samples were transported to a diagnostic microbiology laboratory for processing. Identification of pathogens was usually performed using automated systems such as Vitek-2 (bioMérieux, Marcy-l'Étoile, France), Microscan Walkaway (Beckman Coulter, Brea, CA, USA), or mass spectrometry instruments such as Vitek MS (bioMérieux).^14^ Antimicrobial susceptibility testing was usually performed using Vitek-2 or Microscan, and results were interpreted according to the Clinical and Laboratory Standards Institute recommendations.^14^ Colistin susceptibility results are not reported because the NHLS laboratories did not follow recommended testing methods during the study period.

A line list of culture-positive and culture-negative blood and CSF pathology records from neonates, between Jan 1, 2014, and Dec 31, 2019, inclusive, was extracted from the data warehouse. Data extracted included patient identifying information, demographics, facility name, province in which the facility is located, specimen type, date of specimen collection, tests requested, culture results (organism identification for positive cultures), and antimicrobial susceptibility results. As this study was retrospective and laboratory-based, clinical data such as gestational age, birthweight, peripartum antibiotic exposure, mode of delivery, and HIV status were not available.

### Statistical analysis

Descriptive statistics are presented as medians and interquartile ranges for continuous variables and frequencies and percentages for categorical variables. We calculated the proportion of culture-positive cases by dividing the number of laboratory-confirmed cases (excluding contaminants and duplicates) by the total number of blood culture and CSF specimens (excluding duplicates) submitted per year, and stratified these data by province. We calculated the overall incidence risk by dividing the sum of all cases by the sum of livebirths for the 6-year period. Annual incidence risk of culture-confirmed neonatal BSI and meningitis was calculated by dividing the total number of neonates with culture-confirmed BSI and meningitis in a calendar year by the corresponding annual number of registered livebirths published by Statistics South Africa and expressed per 1000 livebirths.^15^ We further stratified the incidence risk by province. The specimen collection rate was estimated by dividing the total number of all cultures (positive and negative) by the total number of livebirths and expressed per 1000 livebirths. In addition, we stratified the specimen collection rate by province. We used Pearson's chi-squared test and the Wilcoxon rank sum test to determine trends in proportions over time and estimated 95% CIs for proportions.

We calculated the proportion of cases attributed to each of the causative pathogens identified from the pathology records, and stratified these data by syndrome (BSI vs meningitis), hospital tier, and timing of infection. We also calculated the proportions of Gram-positive and Gram-negative bacteria that were susceptible to the various antibiotic regimens identified, and stratified these data by timing of infection and hospital tier.

All statistical analyses were performed using Stata statistical software (version 15).

### Role of the funding source

The funder of the study had no role in study design, data collection, data analysis, data interpretation, or writing of the report.

## Results

**Figure 1:**
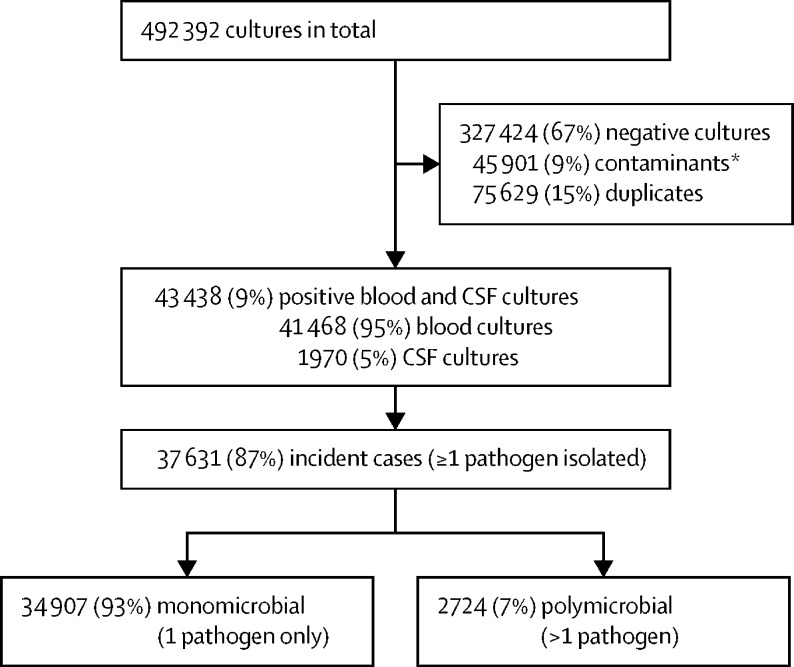
Flow diagram showing the selection of incident neonatal cases of bloodstream infection or meningitis from diagnostic pathology records stored in a national surveillance data warehouse CSF=cerebrospinal fluid. *A contaminant was defined as: (1) coagulase-negative staphylococci (CoNS) isolated from only one specimen, (2) CoNS isolated from two specimens but the second specimen collected >48 h after the first positive culture, or (3) isolation of other common skin commensals considered as blood or CSF specimen contaminants (list of contaminants: *Aerococcus viridans, Bacillus cereus, Corynebacterium* species; CoNS including *Staphylococcus capitis, Staphylococcus cohnii, Staphylococcus epidermidis, Staphylococcus haemolyticus, Staphylococcus hominis, Staphylococcus intermedius, Staphylococcus lentus, Staphylococcus saprophyticus, Staphylococcus urealyticus, Staphylococcus warneri,* other undefined CoNS*;* and *Streptococcus anginosus, Streptococcus gordonii, Streptococcus mitis, Streptococcus oralis, Streptococcus parasanguinis, Streptococcus salivarius, Streptococcus vestibularis, Streptococcus sanguinis*).

During the 6-year period, 492 392 records of neonatal blood and CSF specimens (culture-positive or culture-negative) were extracted. After deduplication to patient level and exclusion of contaminants, 43 438 records of positive cultures (assessed as pathogens) were included in the analysis, with 37 631 defined as incident cases with at least one pathogen isolated (figure 1). This is equivalent to a proportion culture-positive of 9% (95% CI 8·9–9·1) over the 6-year study period (appendix p 6). We observed an overall contamination rate of 9% (45 901 of 492 392 specimens), and this rate remained unchanged over time (p=0·052) during the 6-year period (appendix p 5).

The median age of neonates at the time of diagnosis of an incident culture-confirmed infection was 7 days (IQR 3–14), and 18 397 (49%) were male (table 1). Two-thirds of cases were diagnosed in two provinces: Gauteng and KwaZulu-Natal. The largest number of neonatal cases of BSI and meningitis were diagnosed at regional hospitals (16 630 [44%]), followed by national central hospitals (10 788 [29%]) and provincial tertiary hospitals (6378 [17%]). We classified 6950 (18%) cases as EOS and 30 681 (82%) as LOS.

**Table 1:** Characteristics of neonates with culture-confirmed bloodstream infections and meningitis

		**All patients (n=37 631)**
Age, days		7 (3–14)
Sex		
	Male	18 397 (49%)
	Female	16 331 (43%)
	Unknown	2903 (8%)
Year of diagnosis		
	2014	4986 (13%)
	2015	5832 (16%)
	2016	5879 (16%)
	2017	6269 (19%)
	2018	7227 (19%)
	2019	7438 (20%)
Province in which hospital was located		
	Gauteng	15 432 (41%)
	KwaZulu-Natal	8792 (23%)
	Eastern Cape	3027 (8%)
	Free State	2839 (8%)
	Western Cape	2490 (7%)
	Mpumalanga	1730 (5%)
	North West	1576 (4%)
	Limpopo	1323 (3%)
	Northern Cape	422 (1%)
Hospital category		
	National central hospital	10 788 (29%)
	Provincial tertiary hospital	6378 (17%)
	Regional hospital	16 630 (44%)
	District hospital and others	3835 (10%)
Timing of onset of infection		
	Early-onset neonatal sepsis (0–2 days)	6950 (18%)
	Late-onset neonatal sepsis (3–27 days)	30 681 (82%)

Data are median (IQR) or n (%).

**Figure 2:**
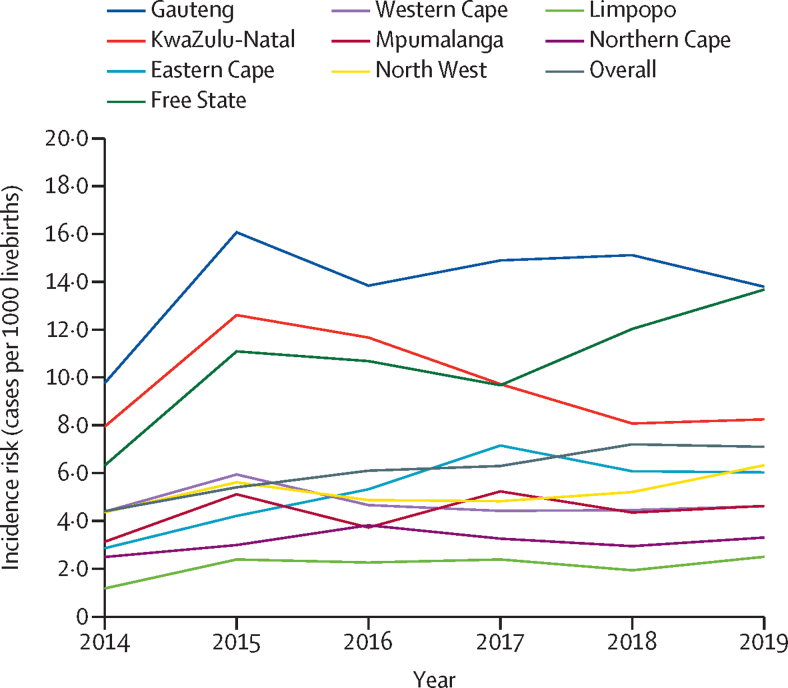
Incidence risk of culture-confirmed bloodstream infection or meningitis among neonates by province (cases per 1000 livebirths)

The overall incidence risk of neonatal BSI and meningitis was 6·0 per 1000 livebirths (95% CI 6·0–6·1) and ranged from 4·4 per 1000 livebirths in 2014 to 7·1 per 1000 livebirths in 2019 (p=0·031; figure 2). The overall incidence risk of LOS was 4·9 per 1000 livebirths (4·9–5·0), and that of EOS was 1·1 per 1000 livebirths (1·1–1·1), with a risk ratio of 4·4 (95% CI 4·3–4·5). In 2019, the incidence risk was highest in the provinces of Gauteng (13·9 per 1000 livebirths [95% CI 13·3–14·7]), Free State (13·7 per 1000 livebirths [12·6–14·7]), and KwaZulu-Natal (8·2 per 1000 livebirths [7·8–8·6]), and lowest in Limpopo (2·5 per 1000 livebirths [2·2–2·8]) and Northern Cape (3·3 per 1000 livebirths [2·6–4·0]; figure 2). Over the 6-year period, the incidence risk increased in Free State province from 6·3 per 1000 livebirths in 2014 (95% CI 5·6–7·0) to 13·7 per 1000 livebirths in 2019 (12·6–14·7), and declined in KwaZulu-Natal province from 12·6 per 1000 livebirths in 2015 (12·0–13·3) to 8·2 per 1000 livebirths in 2019 (7·8–8·6).

Of the 43 438 pathogens isolated from positive blood and CSF cultures, the largest proportion (57%) was Gram-negative bacteria, followed by Gram-positive bacteria (36%) and fungi (7%). Overall, *Klebsiella pneumoniae* (25%) was the most common cause of BSI and meningitis, followed by *Acinetobacter baumannii* (13%) and *Staphylococcus aureus* (12%; table 2). Group B *Streptococcus* (20%), *K pneumoniae* (13%), and *S aureus* (12%) were the most common pathogens among neonates with EOS, whereas *K pneumoniae* (28%), *A baumannii* (14%), and *S aureus* (12%) were the most frequent pathogens causing LOS (appendix p 8). The distribution of pathogens by hospital tier was similar for national central and provincial tertiary hospitals, with Gram-negative bacteria most frequently detected (approximately 60%), followed by Gram-positive bacteria (approximately 30%). In contrast, Gram-positive pathogens (49%) were relatively more common causes of BSI and meningitis at district hospitals, with a similar proportion of Gram-negative bacterial pathogens (48%; table 3). Fungal pathogens, principally *Candida parapsilosis* and *Candida albicans*, were most commonly detected as a cause of invasive neonatal infection at national central hospitals (11%).

**Table 2:** Pathogens isolated from neonates with culture-confirmed bloodstream infections and meningitis

		**Bloodstream and CSF isolates (n=43 438)**	**Bloodstream isolates (n=41 468)**	**CSF isolates (n=1970)**
Gram-negative bacteria		24 836 (57%)	23 689 (57%)	1147 (58%)
	*Klebsiella pneumoniae*	11 155 (26%)	10 885 (26%)	270 (14%)
	*Acinetobacter baumannii*	5686 (13%)	5237 (13%)	449 (23%)
	*Escherichia coli*	2496 (6%)	2336 (6%)	160 (8%)
	*Serratia marcescens*	1446 (3%)	1396 (3%)	50 (3%)
	*Enterobacter cloacae*	1319 (3%)	1245 (4%)	74 (4%)
	*Pseudomonas aeruginosa*	631 (1%)	602 (2%)	29 (1%)
	Other Gram-negative pathogens	2103 (5%)	1988 (5%)	16 (1%)
Gram-positive bacteria		15 595 (36%)	14 823 (36%)	772 (39%)
	*Staphylococcus aureus*	5218 (12%)	5148 (12%)	70 (4%)
	*Enterococcus faecium*	3434 (8%%)	3282 (8%)	152 (8%)
	*Enterococcus faecalis*	3145 (7%)	3037 (7%)	108 (5%)
	Coagulase-negative staphylococci	565 (1%)	494 (1%)	71 (4%)
	Group B *Streptococcus*	2495 (6%)	2135 (5%)	360 (18%)
	Other Gram-positive pathogens	738 (2%)	757 (2%)	11 (1%)
Fungi		3007 (7%)	2956 (7%)	51 (3%)
	*Candida parapsilosis*	1014 (2%)	1009 (2%)	5 (1%)
	*Candida albicans*	965 (2%)	957 (2%)	8 (1%)
	*Candida auris*	60 (0%)	59 (0%)	1 (0%)
	Other yeasts	968 (2%)	931 (2%)	37 (2%)

Data are n (%). CSF=cerebrospinal fluid.

**Table 3:** Pathogens isolated from neonates with culture-confirmed bloodstream infections and meningitis by hospital tier

		**National central (n=13 366)**	**Provincial tertiary (n=7212)**	**Regional (n=18 599)**	**District (n=4261)**
Gram-negative bacteria		8268 (62%)	4638 (64%)	9890 (53%)	2040 (48%)
	*Klebsiella pneumoniae*	3745 (28%)	2221 (31%)	4302 (23%)	887 (21%)
	*Acinetobacter baumannii*	2394 (18%)	1008 (14%)	2010 (11%)	274 (6%)
	*Escherichia coli*	667 (5%)	386 (5%)	1124 (6%)	319 (7%)
	*Serratia marcescens*	465 (3%)	265 (4%)	613 (3%)	103 (2%)
	*Enterobacter cloacae*	341 (3%)	260 (4%)	561 (3%)	157 (4%)
	*Pseudomonas aeruginosa*	235 (2%)	80 (1%)	268 (1%)	48 (1%)
	Other Gram-negative pathogens	421 (3%)	436 (6%)	1012 (5%)	252 (6%)
Gram-positive bacteria		3668 (27%)	2211 (31%)	7623 (40%)	2093 (49%)
	*Staphylococcus aureus*	1244 (9%)	690 (10%)	2547 (14%)	737 (17%)
	*Enterococcus faecium*	834 (6%)	554 (8%)	1627 (9%)	419 (10%)
	*Enterococcus faecalis*	726 (5%)	521 (7%)	1492 (8%)	406 (10%)
	Coagulase-negative staphylococci	232 (2%)	61 (1%)	203 (1%)	69 (2%)
	Group B *Streptococcus*	527 (4%)	317 (4%)	1347 (7%)	304 (7%)
	Other Gram-positive pathogens	105 (1%)	757 (10%)	407 (2%)	158 (4%)
Fungi		1430 (11%)	363 (5%)	1086 (7%)	128 (3%)
	*Candida parapsilosis*	599 (4%)	98 (1%)	305 (2%)	12 (1%)
	*Candida albicans*	442 (3%)	148 (2%)	320 (2%)	55 (1%)
	*Candida auris*	28 (0%)	1 (0%)	31 (0%)	0
	Other yeasts	361 (3%)	116 (2%)	430 (3%)	61 (1%)

Data are n (%).

**Figure 3:**
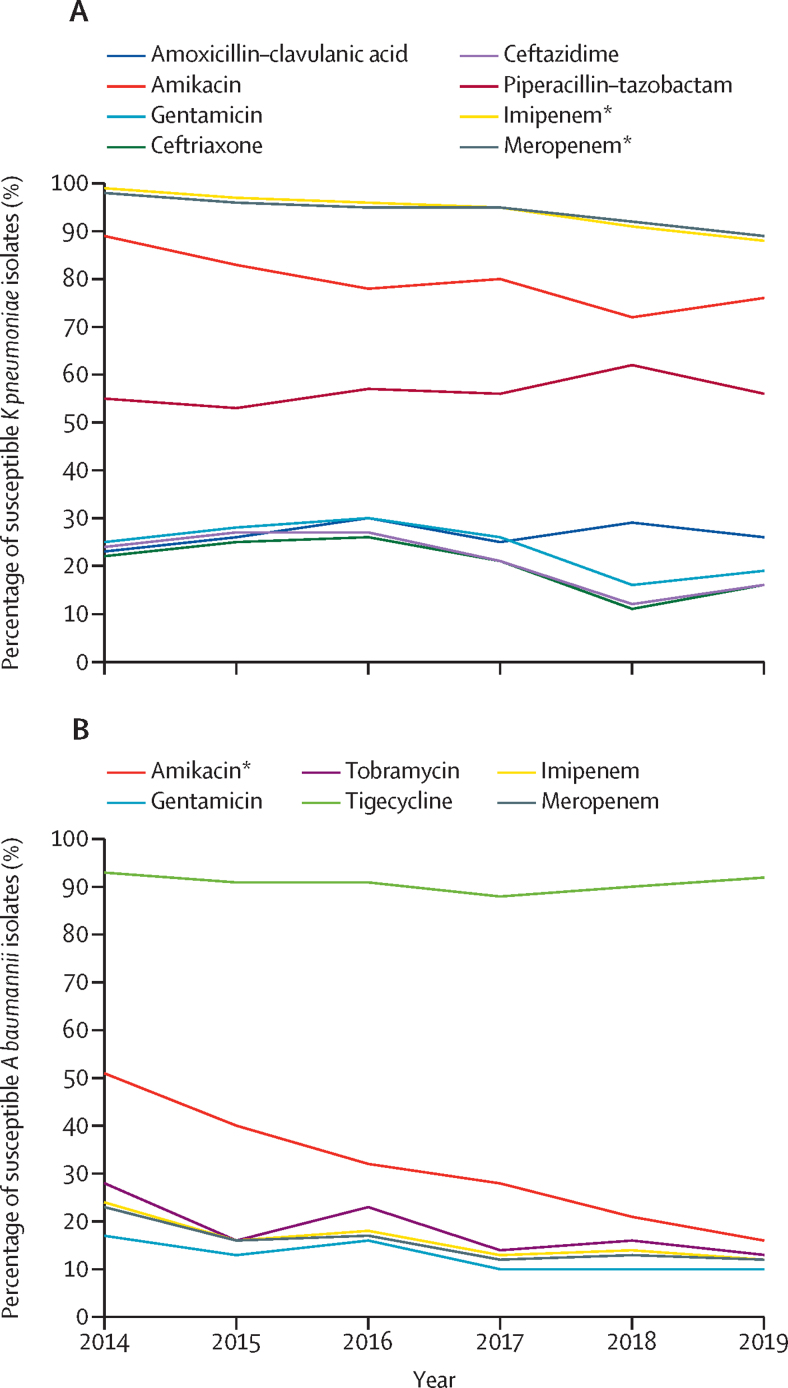
Antimicrobial susceptibility of *Klebsiella pneumoniae* and *Acinetobacter baumannii* isolates among neonates with culture-confirmed bloodstream infection or meningitis (A) *Klebsiella pneumoniae.* (B) *Acinetobacter baumannii.* *p<0·05.

Between 2014 and 2019, there was a substantial reduction in susceptibility of *K pneumoniae* isolates to amikacin (89% to 76%; p=0·051), imipenem (99% to 88%; p=0·030), and meropenem (98% to 89%; p=0·033). *A baumannii* isolates had declining susceptibility over the same period to meropenem (23% to 12%; p=0·051), imipenem (24% to 12%; p=0·054), and amikacin (51% to 16%; p=0·027). However, susceptibility to tigecycline remained above 85% (figure 3 and appendix p 9). A multidrug-resistant phenotype was observed among 8620 (77%) of 11 155 *K pneumoniae* isolates and 5115 (90%) of 5686 of *A baumannii* isolates. Overall, we observed reduced susceptibility of Gram-negative pathogens to ampicillin plus gentamicin, third-generation cephalosporins, piperacillin–tazobactam, and carbapenems (appendix pp 10–13).

The antimicrobial coverage of the WHO-recommended empirical antibiotic regimen for EOS pathogens (ie, ampicillin plus gentamicin) declined from 64% in 2014 to 49% in 2019 (p=0·044). The susceptibility of LOS pathogens to piperacillin–tazobactam plus amikacin declined from 82% to 61% (p=0·030), to imipenem declined from 84% to 66% (p=0·029), and to meropenem declined from 84% to 67% (p=0·028; appendix pp 10–13). We observed a significant reduction in susceptibility of Gram-negative pathogens at provincial tertiary and regional hospitals to the piperacillin–tazobactam plus amikacin regimen and carbapenem regimens (p values of <0·05 for trend analyses). The overall reduced susceptibility to carbapenems was largely attributed to carbapenem-resistant *A baumannii* rather than carbapenem-resistant Enterobacterales. The observed emergence of carbapenem-resistant Enterobacterales in the most recent year of surveillance (ie, 2019) was notable.

## Discussion

We analysed blood and CSF culture pathology records from the public sector in South Africa to obtain a nationwide representative estimate of the burden of culture-confirmed neonatal BSI and meningitis. There was a substantial increase in the national annual incidence risk of invasive neonatal infections over the study period with some provincial variation. During the 6-year period, the annual proportion of positive cultures remained unchanged, while the specimen collection rate increased. The aetiology of neonatal infection differed by syndrome (BSI *vs* meningitis), timing of onset of infection, and hospital level of care, with *K pneumoniae, A baumannii*, and *S aureus* being the most common pathogens. We found declining susceptibility of Gram-negative bacteria to empirical antibiotic regimens over the surveillance period, with reduced susceptibility to carbapenems driven by the emergence of carbapenem-resistant *A baumannii*.

The overall incidence risk of culture-confirmed neonatal BSI and meningitis observed in South Africa was similar to population-based incidence reported in the USA (4·5 to 9·7 per 1000 livebirths) between 1995 and 2005, and in the UK (6·1 per 1000 livebirths) between 2005 and 2014.^16^, ^17^ However, the overall province-specific incidence risks varied in South Africa, with the highest infection risk being 13·7 per 1000 livebirths. Due to the high observed contamination rates for both blood cultures and CSF, we are uncertain if this is a true incidence or if the high contamination rates have masked an even higher incidence risk of BSI and meningitis. We cannot directly compare our estimated national incidence with population-based studies in other low-income and middle-income countries because in those studies BSI and meningitis were defined using clinical criteria and not culture-confirmed.^6^, ^18^

The national incidence of culture-confirmed EOS estimated in our study was relatively high at 1·1 per 1000 livebirths compared with the incidence reported from the UK (0·8 per 1000 livebirths) and the USA (0·8 per 1000 livebirths).^19^, ^20^ However, South Africa's overall national incidence was much lower than reported from a cohort study in Bangladesh (77 per 1000 livebirths, [reported as 12·8% of n=600]) and a single-centre study at a tertiary academic hospital in Soweto, South Africa (3·2 per 1000 livebirths).^21^, ^22^ The incidence risk of culture-confirmed LOS (4·9 per 1000 livebirths) in our study was considerably higher than reported from a Swiss cohort study (0·86 per 1000 livebirths) and also higher than the rate observed in the aforementioned tertiary academic hospital in Soweto (4 per 1000 livebirths).^19^, ^23^ This observation could possibly be ascribed to higher rates of preterm delivery and low birthweights, with consequently longer hospital stays during which horizontal transmission of pathogens occurs in overcrowded units with relatively poor adherence to infection prevention and control measures, all of which increase neonatal risk for developing LOS.

In the present study, Gram-negative bacteria, especially *K pneumoniae* and *A baumannii*, were the dominant pathogens causing BSI and meningitis. *K pneumoniae* has been reported as the leading cause of neonatal infections at tertiary hospitals in South Africa, Botswana, and Greece.^7^, ^24^, ^25^ High rates of BSI caused by multidrug-resistant Gram-negative bacteria in hospital settings are related to suboptimal infection prevention and control measures and inadequate antimicrobial stewardship. Although many studies have reported Group B *Streptococcus* as the sole major pathogen responsible for EOS, we found that Group B *Streptococcus* and *K pneumoniae* were codominant pathogens. Group B *Streptococcus* is usually transmitted vertically from the mother during childbirth, whereas *K pneumoniae* EOS can be a consequence of either horizontal transmission in a contaminated hospital environment or vertical transmission from a mother colonised with *K pneumoniae*. We calculated an early-onset Group B *Streptococcus* incidence risk of 0·3 cases per 1000 livebirths in 2017, which is similar to a published overall estimate of 0·49 cases per 1000 livebirths from a recent systematic review and meta-analysis, but is lower than the estimate of 1·12 cases per 1000 livebirths for Africa in the same study.^26^ This difference might be related to a different study population (infants *vs* neonates) or a laboratory case detection bias.

We found differences in the causative pathogens of laboratory-confirmed BSI and meningitis by hospital tier. Gram-negative bacteria dominated in national central and provincial tertiary hospitals, while Gram-positive bacteria were relatively more frequent in regional and district hospitals. No studies have reported on the causative pathogens of invasive neonatal infection in lower tiers of care in South Africa, but studies from larger hospitals in South Africa confirm the dominance of Gram-negative bacteria.^7^, ^27^ National central and provincial tertiary hospitals have access to antibiotics that district hospitals do not. Therefore, the differences in pathogen distribution by hospital level could be due to differences in antibiotic-prescribing practices resulting in selection of more resistant pathogens at higher tiers. These differences could also be related to the type of care provided at national central and provincial hospitals—eg, neonatal surgery and care of extremely preterm neonates, which could contribute to higher infection rates and antimicrobial resistance. The differences could also be explained by sampling practices at regional and district hospitals, with more invasive BSI and meningitis undiagnosed due to suboptimal blood and CSF collection. However, we think that the variations are less likely to be due to laboratory detection methods because NHLS microbiology laboratories used standardised diagnostic methods. Laboratories with no or limited microbiological capacity referred specimens to larger central laboratories for processing. Although invasive fungal infections were less common overall, the proportion was higher among neonates with LOS and in higher-tier hospitals. *C parapsilosis* was the most common implicated fungal species. Previous South African studies have highlighted the importance of *C parapsilosis* in neonates with high rates of azole resistance.^28^, ^29^ The higher proportion of neonatal *Candida* infections in national central and provincial tertiary hospitals could be caused by detected or undetected outbreaks.^30^

The resistance of *K pneumoniae* to commonly used antibiotics increased from 2014 to 2019, with β-lactam resistance most likely mediated by extended-spectrum β-lactamases. Of concern was the reduced susceptibility of *A baumannii* isolates to carbapenems, which limits the therapeutic options generally to only tigecycline and colistin. There are insufficient pharmacokinetic data for colistin and thus this agent is not currently approved for use in neonates, although it is widely used off-label.^4^ Lack of drug dosing and scheduling recommendations for neonates also makes it difficult to use colistin safely and effectively, and raises concerns about possible emergence of resistance due to suboptimal dosing.^4^

Of particular concern was the low susceptibility of EOS bacterial pathogens to the WHO-recommended first-line empirical regimen ampicillin and gentamicin (also recommended by the South African standard treatment guidelines), with an estimated coverage of only 49% in 2019.^3^, ^31^ We also observed decreasing susceptibility of bacterial pathogens causing LOS over the years. Empirical regimens for EOS and LOS should thus preferably be based on facility-level or unit-level antibiograms. In the absence of such antibiograms or clinical microbiology support, clinicians should conduct unit-specific surveillance to monitor neonatal infections and antimicrobial susceptibility patterns.

Based on a preliminary analysis of these data, a national neonatal sepsis task force was launched in South Africa to convene health professional stakeholders, to raise awareness and advocate to policy makers, and to take active steps to reduce the burden of neonatal infections and related mortality.^32^ In addition, the following steps or actions are planned or already underway in South Africa: (1) the development of a national neonatal infection facility-level dashboard to allow public-sector neonatal units to benchmark their performance on blood culture contamination and infection rates; (2) weighted-incidence syndromic combination antibiogram (WISCA) analytical modelling of the appropriateness of the WHO first-line and second-line regimens using this national dataset; (3) an analysis of factors associated with deaths at neonatal units at provincial tertiary and regional hospitals in South Africa designated as sentinel surveillance sites; and (4) a detailed genome-level characterisation of invasive bacterial and fungal pathogens at the same sentinel surveillance sites.^33^ These data will also serve as a baseline for monitoring the effectiveness of future national and facility-level policies and interventions for infection prevention and control.

Our study had several limitations with respect to the culture-confirmed neonatal BSI and meningitis incidence risk estimates. First, we calculated incidence risk using livebirths as a denominator, thus underestimating the burden of neonatal infection when compared with using patient bed-days as a denominator, which includes only hospitalised neonates, who are at much higher risk of infection. Second, our laboratory dataset did not include gestational age or birthweight of the neonates. Therefore, we were unable to appropriately age-correct for neonates born before 37 weeks’ gestation or those weighing less than 1500 g, and were unable to disaggregate cases among preterm and term babies. Third, we used laboratory definitions, which excluded neonates with culture-negative infections or those who died before a specimen could be collected. With low sensitivity, blood and CSF cultures miss many true cases of infection, especially because the volume of specimens collected from neonates is generally low.^22^, ^33^ In South Africa, many neonates are also initially treated empirically for infections, with no initial cultures being performed. Using a laboratory-based case definition would therefore underestimate the burden of infections. A recent study at a tertiary hospital in South Africa found that about two-thirds of patients eligible for blood culture did not receive one.^34^ Finally, we considered coagulase-negative staphylococci isolated from only one sample as a contaminant, which might have led to an underestimation of the number of infections caused by coagulase-negative staphylococci. The high contamination rate of 9% in our study might also be due to suboptimal specimen collection or limited capacity of diagnostic pathology laboratories to detect, identify, and characterise pathogens, especially in smaller satellite laboratories without on-site pathologists.

In summary, this is the first national population-level analysis of invasive neonatal infections in the South African public health sector. Although our analysis was limited to culture-confirmed infections, we found a high and rising incidence risk of neonatal BSI and meningitis, a predominance of infections caused by *K pneumoniae*, a varying pathogen distribution at different levels of health care, and reduced susceptibility of Gram-negative bacteria to most agents. Our study fills an important knowledge gap, and will serve as a baseline to measure the impact of future interventions in South Africa. Our findings highlight the need to strengthen infection prevention and control measures, antimicrobial stewardship programmes in neonatal units, and appropriate distribution of resources to the different tiers of hospital care.

## Data sharing

Analysis of the data for primary study objectives is planned to be completed latest December, 2025. Additional WISCA analytical modelling will be concluded in December, 2022. Data used for this Article (tables, figures, and supplementary material) after deidentification will be made publicly available no later than 2025. However, if other researchers wish to request access to these data or require additional information, they should communicate with the corresponding author. For data requests, a data sharing agreement will need to be signed between the National Institute for Communicable Diseases and the requestor.

## Declaration of interests

CC and AvG received grant funds from Sanofi Pasteur. AD is supported by a National Institutes of Health Emerging Global Leader Award (NIH K43 TW010682). NPG is partly supported by a National Institutes of Health grant (1R01AI118511-01A1). All other authors declare no competing interests.
