# Supplementary material for: Culture-confirmed neonatal bloodstream infections and meningitis in South Africa, 2014–19: a cross-sectional study
Source: Lancet Glob Health. 2022 Jul 12;10(8):e1170–8. doi: 10.1016/S2214-109X(22)00246-7 (PMC9296659; doi:10.1016/S2214-109X(22)00246-7)
Supplement: Supplementary appendix [file mmc1.pdf]

THE LANCET
Global Health

Supplementary appendix

This appendix formed part of the original submission and has been peer reviewed. We post it as supplied by the authors.

Supplement to: Mashau RC, Meiring ST, Dramowski A, et al. Culture-confirmed neonatal bloodstream infections and meningitis in South Africa, 2014–19: a cross-sectional study. *Lancet Glob Health* 2022; **10:** e1170–78.

Supplementary appendix

Culture-confirmed neonatal bloodstream infections and meningitis in South Africa, 2014 – 2019: a cross-sectional study

Rudzani C. Mashau, MPH[1*]; Susan T. Meiring, MMed[2,3]; Angela Dramowski, PhD[4]; Rindidzani E. Magobo, PhD[1]; Vanessa C. Quan, MMed[2]; Olga Perovic, MMed[1,5]; Anne von Gottberg, PhD[5,6]; Prof Cheryl Cohen, PhD [3,6]; Prof Sithembiso Velaphi, PhD[7]; Erika van Schalkwyk, MMed[1]; Prof Nelesh P. Govender, MMed[1,5,8,9,10] for Baby GERMS-SA

<u>Author affiliations</u>

1. Centre for Healthcare-Associated Infections, Antimicrobial Resistance and Mycoses, National Institute for Communicable Diseases, a Division of the National Health Laboratory Service, 1 Modderfontein Road, Sandringham 2131, Johannesburg, South Africa
2. Division of Public Health Surveillance and Response, National Institute for Communicable Diseases, a Division of the National Health Laboratory Service, 1 Modderfontein Road, Sandringham 2131, Johannesburg, South Africa
3. School of Public Health, Faculty of Health Sciences, University of the Witwatersrand, 7 York Road, Parktown 2193, Johannesburg, South Africa
4. Department of Paediatrics and Child Health, Faculty of Medicine and Health Sciences, Stellenbosch University and Tygerberg Hospital, Francie van Zijl Avenue, Tygerberg 7505, Cape Town, South Africa
5. School of Pathology, Faculty of Health Sciences, University of the Witwatersrand, 7 York Road, Parktown 2193, Johannesburg, South Africa
6. Centre for Respiratory Diseases and Meningitis, National Institute for Communicable Diseases, a Division of the National Health Laboratory Service, 1 Modderfontein Road, Sandringham 2131, Johannesburg, South Africa
7. Department of Paediatrics and Child Health, School of Clinical Medicine, Faculty of Health Sciences, University of the Witwatersrand, 7 York Road, Parktown 2193 and Chris Hani Baragwanath Academic Hospital, 26 Chris Hani Rd, Diepkloof 1864, Johannesburg, South Africa
8. Division of Medical Microbiology, Faculty of Health Sciences, University of Cape Town, Anzio Rd, Observatory 7935, Cape Town, South Africa
9. Institute of Infection and Immunity, St George's University of London, Cranmer Terrace, London SW17 0RE, United Kingdom
10. MRC Centre for Medical Mycology, University of Exeter, Stocker Road Exeter EX4 4QD, United Kingdom

*Corresponding author details: Rudzani Mashau, National Institute for Communicable Diseases, 1 Modderfontein Road, Sandringham 2131, Johannesburg, South Africa. Tel: +27 11 3. E-mail address: rudzanim@nicd.ac.za

Table of Contents

Supplementary methods 3

Supplementary results 4

Supplementary discussion 13

Reference for the Supplementary Appendix 15

Tables and figures appendix

Table S1: General characteristics of hospitals in South Africa by tier 3
Table S2: Number of hospitals by level of facility and province 4
Table S3: Proportion of positive blood and cerebrospinal fluid cultures among neonates 6
Table S4: Pathogens isolated from neonates with culture-confirmed bloodstream infections and meningitis by timing of infection (early-onset: days 0-2 of life versus late-onset: days 3-27) 8
Table S5: Antimicrobial susceptibility of Klebsiella pneumoniae and Acinetobacter baumannii isolates cultured from neonates with bloodstream infections and meningitis 9
Table S6: Antimicrobial susceptibility of bacterial isolates cultured from neonates with bloodstream infections and meningitis in South Africa by broad bacterial group, timing of infection and tier of hospital care 10

Supplementary methods

The NHLS is the sole pathology laboratory service provider for public facilities offering standardised diagnostic methods across the laboratory network. NHLS microbiology laboratories participated in relevant external quality assessment schemes.[1] Approximately 100 NHLS laboratories were accredited with South African National Accreditation System to ISO: 15189 by 2021. During the study period, pathology test results were captured in either of two laboratory information systems used by NHLS laboratories (initially DisaLab then TrakCare) and data from authorised pathology reports were then archived in the data warehouse..[1] The proportion of accredited laboratories differs by level of care and province, with relatively more accredited facilities in Gauteng, Western Cape and KwaZulu-Natal provinces and academic laboratories generally being more likely to be accredited than non-academic laboratories, and for a longer time period.

Hospitals in South Africa are categorised into four tiers (Table S1): 1) national central hospitals, which comprise of highly-specialised referral units, multi-speciality clinical services, innovation and research; 2) provincial tertiary hospitals, the next level of care which receive referrals from and provide sub-specialist support to regional hospitals; 3) regional hospitals, which receive referrals from and provide specialist support to district hospitals; and 4) district hospitals which receive referrals from and provide generalist support to community healthcare centres and primary healthcare clinics.[2] Approximately one million babies are born in South Africa annually. The majority of these babies are born in public-sector hospitals and births are recorded in real-time during the year by the Department of Home Affairs and reported annually. The number of hospital facilities, by level and province, is shown in Table S2.

Table S1: General characteristics of hospitals in South Africa by tier

**General characteristics**	**National central**	**Provincial tertiary**	**Regional**	**District**
**Microbiology laboratory**				
Referral of blood or cerebrospinal fluid (CSF) for processing	Onsite	Onsite	Onsite or offsite	Onsite or offsite
Blood culture processing method	Automated systems	Automated systems	Automated systems	Automated systems
CSF processing method	Manual	Manual	Manual	Manual
Direct microscopy of blood and CSF	+	+	+	+
Culture	Bacterial and fungal	Bacterial and fungal	Bacterial and fungal	Bacterial and fungal
Pathogen identification	Automated systems including mass spectrometry instruments	Automated systems including mass spectrometry	Automated systems	Automated systems
Antimicrobial susceptibility testing	Automated systems	Automated systems	Automated systems	Automated systems
Specimen volumes	++++	+++	++	++
Accreditation to ISO 15189	+++	+++	++	+
**Neonatal and related services**				
Neonatal ICU or high care beds	+	+	-	-
Dedicated neonatal unit	+	+	+	+/-
Neonatologist	+++	++	-	-
Dedicated infection control team for the hospital	++	++	+	+
Obstetric care	High-risk pregnancies with maternity ICU	High-risk pregnancies with maternity ICU	Low-risk pregnancies with no maternity ICU	Low-risk pregnancies with no maternity ICU

Table S2: Number of hospitals by level of facility and province

Province	National central hospitals, n (%)	Provincial tertiary hospitals, n (%)	Regional hospitals, n (%)	District hospitals, n (%)
Gauteng	4 (45)	2 (13)	11 (23)	15 (8)
KwaZulu-Natal	1 (11)	3 (19)	11 (23)	41 (22)
Eastern Cape	1 (11)	1 (6)	4 (8)	25 (14)
Free State	1 (11)	1 (6)	4 (8)	11 (6)
Western Cape	2 (22)	1 (6)	5 (10)	35 (19)
Mpumalanga	-	2 (13)	3 (6)	18 (10)
North West	-	3 (19)	3 (6)	10 (5)
Limpopo	-	2 (13)	6 (13)	25 (14)
Northern Cape	-	1 (5)	1 (1)	4 (2)
**Total**	**9 (4)**	**16 (6)**	**48 (19)**	**184 (71)**

We used the Centres for Disease Control and Prevention (CDC) organism lists to exclude common skin commensals considered as contaminants.[3] The NHLS laboratory information systems did not capture data on the specific anatomical sites from which blood was collected; therefore, this was not used as a criterion to define pathogenic CoNS. For neonates with multiple pathogens isolated within a 14-day period, the first isolation was considered as the date of diagnosis. However, all cultured pathogens were included in the denominator for calculating the prevalence of neonatal infection aetiologies. If the same pathogen was cultured from a subsequent specimen in the same patient after 14 days of the first positive culture, this was considered a recurrent episode. However, if the same pathogen was cultured from a subsequent specimen in the same patient within 14 days of the positive culture, this was considered a duplicate and excluded from the analysis. Where both blood and CSF specimens were positive for the same organism and within 14 days of the first positive culture, the CSF isolate was considered to be the primary positive culture and meningitis was recorded as the diagnosis. We were unable to categorise CoNS with only a single culture as a true pathogen without clinical information (e.g. antimicrobial therapy targeting CoNS) or additional laboratory data (e.g. C-reactive protein levels). Therefore, all CoNS cases with a single cultured isolate were excluded from the analysis and regarded as contaminants.

Supplementary results

In 2019, the most recent year of surveillance, the highest proportion of positive cultures was observed in the Free State (16.9%) and Limpopo (16.1%) provinces, while the lowest proportions were observed in Western Cape (5.4%) and KwaZulu-Natal (6.8%) provinces (Table S3). However, the positive culture yield consistently remained the same over time (Figure S1). We observed an overall contamination rate of 9% (45,901/492,392), and this remained unchanged over time (p-value of 0.05) during the 6-year period (Figure S1).

Figure S1: Trends in positive cultures, and blood and cerebrospinal fluid contamination rates

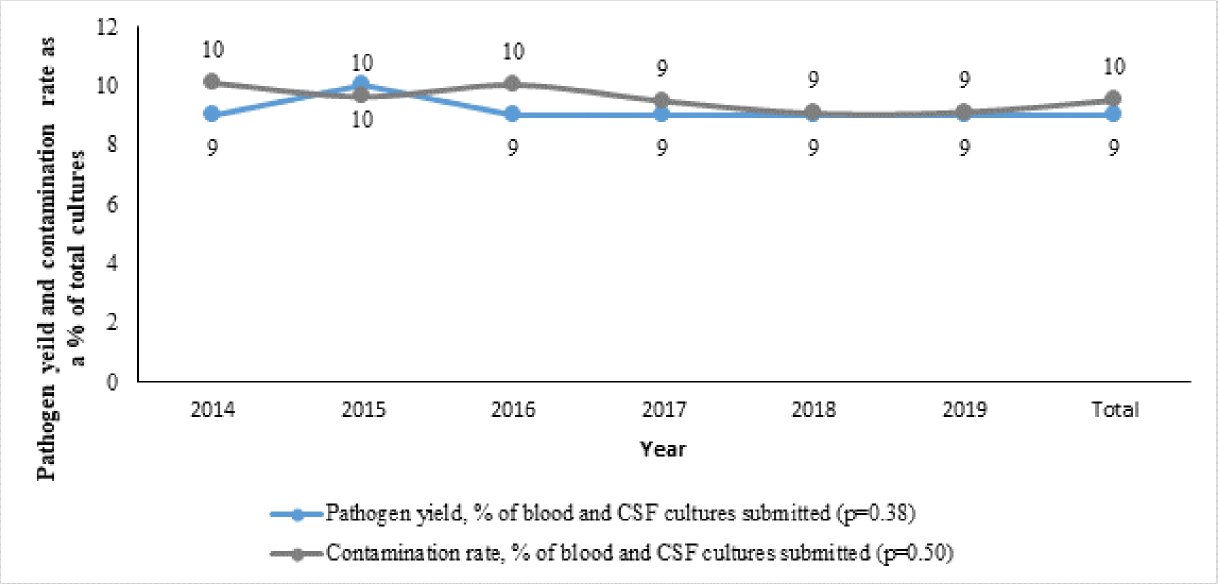

Table S3: Proportion of positive blood and cerebrospinal fluid cultures among neonates

**Province**	**All total n/N (%)**	**2014 n/N (%)**	**2015 n/N (%)**	**2016 n/N (%)**	**2017 n/N (%)**	**2018 n/N (%)**	**2019 n/N (%)**
Gauteng	18208/184207 (10)	2250/22904 (10)	3012/26583 (11)	2827/29221 (10)	3089/33193 (9)	3647/36646 (10)	3383/35660 (10)
KwaZulu-Natal	9824/143372 (7)	1577/21216 (7)	1599/22129 (7)	1666/22987 (7)	1 384/23078 (6)	1738/26793 (7)	1860/27169 (7)
Eastern Cape	3606/29159 (12)	368/1994 (19)	374/2434 (15)	556/3505 (16)	787/6312 (13)	764/6979 (11)	757/7935 (10)
Free State	3489/21722 (16)	362/2799 (13)	552/3204 (17)	562/3512 (16)	545/3623 (15)	698/4037 (17)	770/4550 (17)
Western Cape	2852/58667 (5)	466/10598 (4)	500/10362 (5)	434/8991 (5)	429/9062 (5)	494/9853 (5)	529/9801 (5)
Mpumalanga	1838/18280 (10)	239/2314 (10)	257/2372 (11)	234/2229 (11)	356/3205 (11)	358/3766 (10)	394/4394 (9)
North West	1704/13555 (13)	238/1674 (14)	247/1724 (14)	246/1862 (13)	259/2165 (12)	312/2623 (12)	402/3507 (12)
Limpopo	1469/19793 (15)	140/649 (22)	183/998 (18)	246/1718 (14)	267/1877 (14)	271/2297 (12)	362/2254 (16)
Northern Cape	448/3583 (13)	67/501 (13)	66/468 (14)	87/507 (17)	72/598 (12)	76/679 (11)	80/830 (10)
**South Africa**	**43438/482338 (9)**	**5707/64649 (9)**	**6790/70274 (10)**	**6858/74532 (9)**	**7188/83113 (9)**	**8358/93673 (9)**	**8537/96100 (9)**

Over the entire period, the provincial contribution to the total cases ranged from 422 cases (1% of all cases) in the Northern Cape Province to 15,432 cases (41% of all cases) in Gauteng Province.
The median annual number of blood and CSF culture tests performed during the reporting period was 78,823 (IQR: 68,867 – 94,280). The overall specimen collection rate was 77 per 1000 livebirths (95% CI 77.0-77.4), ranging from 57 per 1000 livebirths in 2014 to 91 per 1000 in 2019 (p=0.03). In 2019, the specimen collection rates were highest in Gauteng (170 per 1000 livebirths), KwaZulu-Natal (136 per 1000 livebirths) and Western Cape provinces (98 per 1000 livebirths), and lowest in Limpopo (18 per 1000 livebirths) and Northern Cape provinces (35 per 1000) [Figure S2].

Figure S2: Blood culture and cerebrospinal fluid specimen collection rate (specimens collected per 1000 live births) by year and province among neonates, 2014 – 2019, South Africa

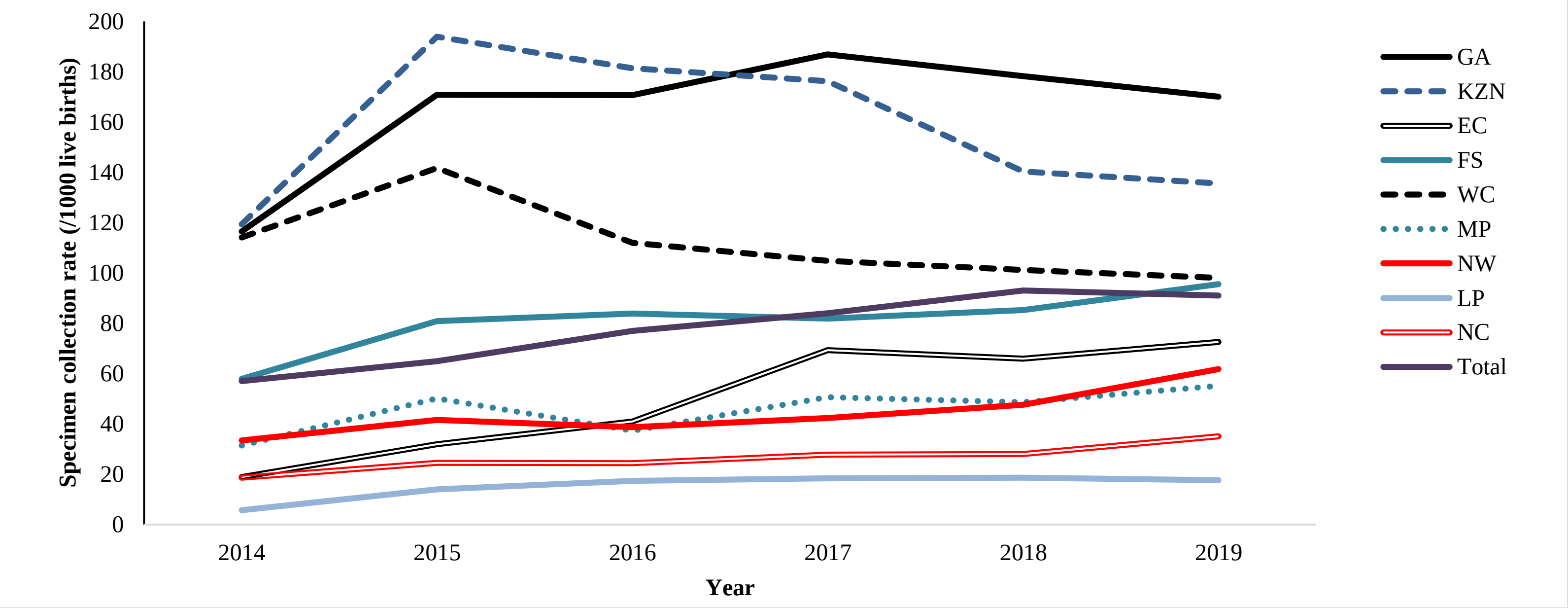

Abbreviations: Gauteng (GP), KwaZulu-Natal (KZN), Eastern Cape (EC), Free State (FS), Western Cape (WC), Mpumalanga (MP), North West (NW), Limpopo (LP), Northern Cape (NC) provinces

Table S4: Pathogens isolated from neonates with culture-confirmed bloodstream infections and meningitis by timing of infection (early-onset: days 0-2 of life versus late-onset: days 3-27)

**Pathogen**	**Early-onset sepsis, n=7,580 n (%)**	**Late-onset sepsis, n=35,858 n (%)**
Gram-negative bacteria	3,333 (44)	21,503 (60)
Klebsiella pneumoniae	956 (13)	10,199 (28)
Acinetobacter baumannii	681 (9)	5,005 (14)
Escherichia coli	624 (8)	1,872 (5)
Serratia marcescens	129 (2)	1,317 (4)
Enterobacter cloacae	266 (4)	1,053 (3)
Pseudomonas aeruginosa	168 (2)	463 (1)
Other Gram-negative pathogens	509 (6.7)	1,597 (4)
Gram-positive bacteria	4,041 (53)	11,554 (32)
Staphylococcus aureus	932 (12)	4,286 (12)
Enterococcus faecium	387 (5)	3,047 (9)
Enterococcus faecalis	889 (11)	2,256 (6)
Coagulase-negative staphylococci	63 (1)	502 (1)
Group B *Streptococcus*	1,498 (20)	997 (3)
Other Gram-positive pathogens	272 (4)	466 (1)
Fungi	206 (3)	2,801 (8)
Candida parapsilosis	36 (1)	978 (3)
Candida albicans	71 (1)	894 (2)
Candida auris	2 (0)	58 (0)
Other yeasts	97 (1)	868 (3)

Table S5: Antimicrobial susceptibility of *Klebsiella pneumoniae* and *Acinetobacter baumannii* isolates cultured from neonates with bloodstream infections and meningitis

Pathogen	Antibiotic	2014	2015	2016	2017	2018	2019	P value
Klebsiella pneumonia	Amoxicillin/clavulanic	279/1193 (23)	400/1511 (26)	485/1620 (30)	405/1637 (25)	646/2221 (29)	547/2144 (26)	0.38
	Amikacin	1089/1222 (89)	1299/1547 (83)	1260/1619 (78)	1315/1649 (80)	1621/2245 (72)	1636/2147 (76)	0.051
	Gentamicin	307/1231 (25)	428/1556 (28)	485/1627 (30)	426/1631 (26)	356/2232 (16)	405/2151 (19)	0.122
	Ceftriaxone	232/1047 (22)	349/1421 (25)	412/1582 (26)	329/1596 (21)	252/2230 (11)	337/2138 (16)	0,107
	Ceftazidime	270/1114 (24)	388/1450 (27)	418/1571 (27)	343/1604 (21)	256/2204 (12)	333/2084 (16)	0.075
	Piperacilin/tazobactam	601/1087 (55)	743/1400 (53)	875/1531 (57)	882/1574 (56)	1356/2205 (62)	1166/2096 (56)	0.219
	Imipenem	1084/1099 (99)	1324/1371 (97)	1463/1525 (96)	1463/1547 (95)	1981/2172 (91)	1857/2100 (88)	0.030
	Meropenem	1114/1132 (98)	1429/1490 (96)	1465/1539 (95)	1510/1592 (95)	2007/2186 (92)	1877/2114 (89)	0.033
Acinetobacter baumannii	Amikacin	231/451 (51)	194/489 (40)	183/579 (32)	174/621 (28)	128/624 (21)	113/693 (16)	0.027
	Gentamicin	79/453 (17)	74/556 (13)	122/761 (16)	94/984 (10)	110/1126 (10)	143/1384 (10)	0.062
	Tobramycin	76/333 (23)	55/353 (16)	101/445 (23)	67/492 (14)	86/535 (16)	75/583 (13)	0.09
	Tigecycline	132/142 (93)	160/175 (91)	310/339 (91)	485/554 (88)	694/768 (90)	895/974 (92)	0.445
	Imipenem	102/417 (24)	87/539 (16)	138/761 (18)	121/952 (13)	154/1137 (14)	161/1372 (12)	0.054
	Meropenem	103/443 (23)	86/553 (16)	130/758 (17)	113/979 (12)	153/1144 (13)	160/1377 (12)	0.051

Table S6: Antimicrobial susceptibility of bacterial isolates cultured from neonates with bloodstream infections and meningitis in South Africa by broad bacterial group, timing of infection and tier of hospital care

Pathogen group	Antibiotic	2014	2015	2016	2017	2018	2019	p-value
**All**								
Gram-negative bacteria	Ampicillin & gentamicin	1119/2768 (40)	1322/3369 (39)	1392/3532 (39)	1379/3905 (35)	1376/4660 (30)	1596/4896 (33)	0.049
	Third generation cephalosporins	1072/2706 (40)	1294/3342 (39)	1371/3552 (39)	1351/3951 (34)	1320/4749 (28)	1534/4920 (31)	0.062
	Piperacillin/tazobactam & amikacin	2263/2723 (83)	2587/3332 (78)	2419/3512 (69)	2579/3906 (66)	2926/4656 (63)	3026/4883 (62)	0.042
	Imipenem	1972/2354 (84)	2272/2851 (80)	2438/3200 (76)	2412/3428 (70)	3030/4313 (70)	3034/4609 (66)	0.028
	Meropenem	2080/2494 (83)	2565/3181 (81)	2549/3327 (77)	2655/3700 (72)	3252/4522 (72)	3256/4815 (68)	0.028
Gram-positive bacteria	Gentamicin	275/502 (55)	307/652 (47)	318/590 (54)	368/594 (62)	442/813 (54)	475/767 (62)	0.12
	Penicillin	607/1332 (46)	674/1535 (44)	670/1551 (43)	783/1645 (48)	935/2058 (45)	969/2088 (46)	0.59
	Cloxacillin	316/589 (54)	376/705 (53)	349/604 (58)	398/638 (62)	454/860 (53)	477/797 (60)	0.30
	Vancomycin	1691/1765 (96)	1985/2074 (96)	2044/2083 (98)	2045/2089 (98)	2475/2511 (99)	2437/2473 (99)	0.19
	Linezolid	1096/1124 (98)	1313/1353 (97)	1489/1497 (99)	1518/1526 (99)	2039/2046 (100)	2072/2089 (99)	0.11
**Early-onset sepsis (0-3 days)**								
Gram-negative bacteria	Ampicillin & gentamicin	240/377 (64)	257/446 (58)	230/401 (57)	278/504 (55)	260/588 (44)	346/702 (49)	0.044
	Third generation cephalosporins	222/364 (61)	259/441 (59)	234/406 (58)	273/504 (54)	272/604 (45)	352/705 (50)	0.047
	Piperacillin/tazobactam & amikacin	311/350 (89)	357/84 (84)	275/386 (71)	346/490 (71)	393/569 (69)	451/689 (65)	0.037
	Imipenem	250/295 (85)	307/369 (83)	285/362 (79)	325/437 (74)	391/531 (74)	437/653 (67)	0.029
	Meropenem	256/312 (82)	343/405 (85)	297/374 (79)	352/466 (76)	429/558 (77)	466/678 (69)	0.049
Gram-positive bacteria	Gentamicin	74/106 (70)	94/142 (66)	74/111 (67)	91/117 (78)	98/128 (77)	100/139 (72)	0.20
	Penicillin	71/73 (97)	92/98 (94)	68/70 (97)	88/96 (92)	92/95 (97)	91/92 (99)	0.51
	Cloxacillin	85/135 (63)	121/169 (72)	91/131 (69)	96/123 (78)	109/165 (66)	95/147 (65)	0.98

	Vancomycin	440/456 (96)	562/572 (98)	517/524 (99)	552/559 (99)	609/620 (98)	650/659 (99)	0.13
	Linezolid	238/242 (98)	303/308 (98)	305/308 (99)	363/365 (99)	451/453 (100)	500/505 (99)	0.17
**Late-onset sepsis (3-27 days)**								
Gram-negative bacteria	Ampicillin & gentamicin	879/2391 (37)	1065/2923 (36)	1162/3131 (37)	1101/3401 (32)	1116/4072 (27)	1250/4193 (30)	0.06
	Third generation cephalosporins	850/2342 (36)	1035/2901 (36)	1137/3146 (36)	1078/3447 (31)	1048/4145 (25)	1182/4215 (28)	0.051
	Piperacillin/tazobactam & amikacin	1952/2373 (82)	2230/2908 (77)	2144/3126 (69)	2233/3416 (65)	2533/4087 (62)	2575/4194 (61)	0.030
	Imipenem	1722/2059 (84)	1965/2482 (79)	2153/2838 (76)	2087/2991 (70)	2639/3782 (70)	2597/3956 (66)	0.029
	Meropenem	1824/2182 (84)	2222/2776 (80)	2252/2953 (76)	2303/3234 (71)	2823/3964 (71)	2790/4137 (67)	0.028
Gram-positive bacteria	Gentamicin	201/396 (51)	213/510 (42)	244/479 (51)	277/477 (58)	344/685 (50)	375/628 (60)	0.16
	Penicillin	299/702 (43)	313/786 (40)	386/922 (42)	412/876 (47)	488/1055 (46)	511/1089 (47)	0.079
	Cloxacillin	231/454 (51)	255/536 (48)	258/473 (55)	302/515 (59)	345/695 (50)	382/650 (59)	0.21
	Vancomycin	1251/1309 (96)	1423/1502 (95)	1527/1559 (98)	1493/1530 (98)	1866/1891 (99)	1787/1814 (99)	0.20
	Linezolid	858/882 (97)	1010/1045 (97)	1184/1189 (100)	1155/1161 (99)	1588/1593 (100)	1572/1584 (99)	0.19
**National central hospitals**								
Gram-negative bacteria	Ampicillin & gentamicin	303/794 (38)	519/1239 (42)	499/1249 (40)	434/1357 (32)	363/1717 (21)	396/1595 (25)	0.057
	Third generation cephalosporins	331/800 (41)	535/1243 (43)	540/1270 (43)	501/1394 (36)	383/1766 (22)	456/1610 (28)	0.065
	Piperacillin/tazobactam & amikacin	602/783 (77)	902/1227 (74)	844/1251 (67)	886/1375 (64)	944/1747 (54)	821/1599 (51)	0.027
	Imipenem	531/729 (73)	853/1176 (73)	833/1210 (69)	814/1314 (62)	1162/1671 (70)	972/1547 (63)	0.10
	Meropenem	560/774 (72)	905/1234 (73)	863/1236 (70)	851/1354 (63)	1219/1733 (70)	1024/1588 (64)	0.11
Gram-positive bacteria	Gentamicin	64/168 (38)	70/196 (36)	77/170 (45)	84/144 (58)	88/199 (44)	63/135 (47)	0.21
	Penicillin	123/314 (39)	138/376 (37)	141/357 (40)	138/323 (43)	189/446 (42)	167/386 (43)	0.061
	Cloxacillin	56/150 (37)	72/180 (40)	77/155 (50)	82/143 (57)	84/183 (46)	62/129 (48)	0.18
	Vancomycin	467/473 (99)	530/545 (97)	524/532 (99)	477/486 (98)	575/581 (99)	513/520 (99)	0.48
	Linezolid	428/431 (99)	467/476 (98)	456/456 (100)	367/370 (99)	505/507 (100)	445/447 (100)	0.14
**Provincial tertiary hospitals**								

Gram-negative bacteria	Ampicillin & gentamicin	286/704 (41)	210/692 (30)	234/691 (34)	189/670 (28)	221/781 (28)	281/898 (31)	0.13
	Third generation cephalosporins	254/695 (37)	188/683 (28)	216/693 (31)	181/674 (27)	229/797 (29)	252/908 (28)	0.14
	Piperacillin/tazobactam & amikacin	565/692 (82)	536/691 (78)	485/688 (70)	439/673 (65)	525/776 (68)	569/907 (63)	0.038
	Imipenem	510/590 (86)	455/548 (83)	464/580 (80)	385/522 (74)	447/671 (67)	555/848 (65)	0.027
	Meropenem	524/608 (86)	475/577 (82)	444/567 (78)	402/549 (73)	479/704 (68)	589/889 (66)	0.026
Gram-positive bacteria	Gentamicin	28/59 (47)	27/54 (50)	27/53 (51)	65/86 (76)	62/105 (59)	89/133 (67)	0.11
	Penicillin	74/196 (38)	76/205 (37)	83/211 (39)	116/267 (43)	133/284 (47)	150/335 (45)	0.044
	Cloxacillin	44/88 (50)	60/85 (71)	45/71 (63)	79/98 (81)	71/119 (60)	88/134 (66)	0.46
	Vancomycin	304/331 (92)	294/324 (91)	284/291 (98)	302/314 (96)	335/341 (98)	362/365 (99)	0.057
	Linezolid	129/132 (98)	161/169 (95)	181/182 (99)	221/221 (100)	263/265 (99)	321/323 (99)	0.22
**Regional hospitals**								
Gram-negative bacteria	Ampicillin & gentamicin	432/1101 (39)	499/1229 (41)	546/1377 (40)	637/1564 (41)	618/1769 (35)	665/1852 (36)	0.14
	Third generation cephalosporins	400/1047 (38)	479/1207 (40)	502/1376 (36)	553/1565 (35)	547/1791 (31)	596/1853 (32)	0.044
	Piperacillin/tazobactam & amikacin	939/1081 (87)	973/1212 (80)	918/1364 (67)	1023/1544 (66)	1140/1751 (65)	1201/1835 (65)	0.049
	Imipenem	790/892 (89)	792/948 (84)	962/1216 (79)	972/1316 (74)	1103/1622 (68)	1072/1705 (63)	0.025
	Meropenem	843/955 (88)	996/1175 (85)	1046/1315 (80)	1137/1496 (76)	1213/1715 (71)	1170/1795 (65)	0.026
Gram-positive bacteria	Gentamicin	141/221 (64)	172/342 (50)	162/295 (55)	157/282 (56)	197/378 (52)	214/352 (61)	0.86
	Penicillin	341/732 (47)	387/832 (47)	385/848 (45)	422/839 (50)	463/1042 (44)	489/530 (48)	0.96
	Cloxacillin	162/283 (57)	189/360 (53)	167/299 (56)	175/313 (56)	205/421 (49)	228/390 (58)	0.80
	Vancomycin	755/793 (95)	962/1000 (96)	1009/1029 (98)	963/979 (98)	1178/1193 (99)	1123/1143 (98)	0.057
	Linezolid	449/469 (96)	589/610 (97)	695/700 (99)	744/747 (100)	986/987 (100)	971/981 (99)	0.69
**District hospitals**								
Gram-negative bacteria	Ampicillin & gentamicin	98/169 (58)	94/209 (45)	113/215 (53)	119/314 (38)	174/393 (44)	254/550 (46)	0.19
	Third generation cephalosporins	87/164 (53)	92/209 (44)	113/213 (53)	116/318 (36)	161/395 (41)	230/549 (42)	0.16
	Piperacillin/tazobactam & amikacin	157/167 (94)	176/202 (87)	172/209 (82)	231/314 (74)	317/382 (83)	435/542 (80)	0.11
	Imipenem	141/143 (99)	172/179 (96)	176/194 (92)	241/276 (87)	318/349 (91)	435/509 (85)	0.042

	Meropenem	153/157 (97)	189/195 (97)	196/209 (94)	265/301 (88)	341/370 (92)	473/543 (87)	0.050
Gram-positive bacteria	Gentamicin	42/54 (78)	38/60 (63)	52/72 (72)	62/82 (76)	95/131 (73)	109/147 (74)	0.75
	Penicillin	71/149 (48)	75/169 (44)	63/177 (36)	111/249 (45)	151/333 (45)	164/386 (42)	0.60
	Cloxacillin	54/68 (79)	55/80 (69)	60/79 (76)	62/84 (74)	94/137 (69)	99/144 (69)	0.15
	Vancomycin	165/168 (98)	199/205 (97)	227/231 (98)	303/310 (98)	387/396 (98)	439/445 (99)	0.13
	Linezolid	90/92 (98)	96/98 (98)	157/159 (99)	186/188 (99)	285/287 (99)	335/338 (99)	0.064

Supplementary discussion

In the most recent year studied (2019), the province-specific incidence risk was highest in Gauteng, Free State and KwaZulu-Natal provinces, and lowest in Limpopo and Northern Cape provinces. The differences in provincial incidence may be due to the geographical location of national central and provincial tertiary hospitals; hospitals with high incidence rates have large neonatal units providing higher levels of care (with a higher frequency of invasive medical procedures and neonatal surgery) leading to increased risk of neonatal infection. Alternatively, the difference in incidence could be due to differences in specimen collection rates. In our study, with the exception of the Western Cape Province, provinces with a high incidence also had high specimen collection rates, suggesting that the low incidence in some provinces could be an underestimation of the burden of disease due to inadequate specimen collection.

Although the number of live births remained relatively stable (increased/decreased by <2%), we observed an overall increase in the specimen collection rate. This may indicate an increasing need among clinicians to perform diagnostic investigation workups for clinically-suspected HAIs in overcrowded neonatal units during suspected infection outbreaks. Alternatively, this may reflect increased neonatal care resources which allows for more low-gestational age babies to survive for longer periods in hospital and increases their time at risk for HAIs.

Although, specimen testing rates increased, the blood culture contamination rate was 3 times higher than the international best practice norm of <3%.[4] Our definition for assigning "pathogenic" status to usual contaminants may have been too restrictive and some single positive blood cultures may have represented true episodes of neonatal infection.[5] In addition, contaminants may have overgrown the true pathogens, thereby leading to misclassification of true infections as contaminants. The high contamination rate suggests insufficient blood volume sampling in new-born babies, poor adherence to skin antisepsis practices at the time of blood culture collection and/or non-adherence to specimen transportation recommendations.[6]

Case ascertainment issues may have biased our estimate of the national incidence of neonatal BSI and meningitis in South Africa for a number of reasons listed below.

1. Care-seeking gaps: We did not account for neonates who died at home shortly after birth with possible neonatal BSI/ meningitis before care could be sought and samples could be collected.
2. Coverage and quality of care gaps: Quality of care may have varied across the South African health service and thus not all neonates with invasive infection may have been recognised or diagnosed, especially since the signs of sepsis may be subtle.
3. Diagnostics gap:
    a. Specimen-taking practices for invasive neonatal infections may have varied by hospital level, e.g. district versus national central, even among neonates who were born in hospital or later admitted to hospital and recognised to have signs of sepsis.
    b. Since detailed clinical information was not available in this laboratory dataset (e.g. peripartum antibiotic exposure prior to collection of samples for culture), we were unable to assess the effect of such factors on culture positivity. However, since culture-positive infections are well-recognised to represent only a fraction of all cases, we certainly would have underestimated the true burden of invasive infections.
4. Diagnostic laboratory gaps:
    a. Clinical microbiological practices may have varied by level of laboratory and this could potentially account for under-detection as well as variations in pathogens and AST results reported. Confirmatory AST methods may not have been performed or recorded for some pathogens consistently. For example, vancomycin resistance for *S. aureus* requires confirmatory testing, which may not have been available at routine laboratories.
    b. Undetected clusters or outbreaks may have also impacted on the number of recorded cases as well as the reported pathogen and antimicrobial resistance distribution.
5. Laboratory surveillance gaps:
    a. We included only positive cultures of neonates who were seen in the public healthcare sector, which serves an estimated 80% of the South African population, and omitted cultures from neonates at private facilities which admit patients with health insurance. Therefore, it is likely that the overall burden of BSI and meningitis was underestimated. However, we believe that neonatal infections, particularly healthcare-associated infections, occur at a somewhat lower frequency in the private versus the public sector.

b. We excluded laboratory records with a missing date of birth or those with missing age and with a ward name not suggestive of a neonatal unit/ nursery/ kangaroo mother care. Therefore, we may have under-counted true neonatal cases.

c. To identify unique neonates from laboratory records, a probabilistic linking-algorithm was applied by the data warehouse, using a number of available patient identifiers. In a study of viral load records, this algorithm was found to have a sensitivity of 73% and positive predictive value of 83% for adult patients. However, the performance is unknown for neonates who may not have a recorded first name early in life and were usually registered using their mother's names (often named 'baby of' [mother's name] [mother's surname]). As a result, we may have overestimated the incidence of culture-confirmed BSI and meningitis because neonates with multiple tests under different names may have been counted more than once.

d. We applied an arbitrary definition to exclude subsequent positive cultures (i.e. 14 days following the first positive culture). Some of these may have represented new infection episodes. This was done due to a lack of clinical and treatment information but as a result, we may have under-counted neonatal infection episodes."

e. Finally, our data extract from the data warehouse excluded cases with laboratory-confirmed *Listeria monocytogenes* owing to an ongoing class action lawsuit related to a large South African outbreak.[7,8] This may have resulted in the underestimation of the overall incidence as *L. monocytogenes* is a relatively common neonatal infection in South Africa. During the outbreak period from June 2017 to April 2018, 406 neonatal cases of listeriosis were reported.[7]

Reference for the Supplementary Appendix

1. National Health Laboratory Service (NHLS). National Health Laboratory Service Annual Report 2018/19. 2019; Available from: https://www.nhls.ac.za/wp-content/uploads/2020/05/NHLS_Annual_Report_2019_Revised_00000004.pdf (accessed April 20,2022).
2. South Africa Department of Health. Referral System: Levels of Health Care. 2014. Available from: http://www.kznhealth.gov.za/Referral-system.htm/ (accessed May 19, 2021).
3. Centers for Disease Control and Prevention and the National Healthcare Safety Network. CDC/NHSH Bloodstream Infection Event (Central Line-Associated Bloodstream Infection and Non-central Line-associated Bloodstream Infection). 2015. Available from: https://www.cdc.gov/nhsn/xls/2018-NHSN-Organisms-List-Validation.xlsx (accessed April 20, 2022).
4. Mudzikati L, Dramowski A. Neonatal septicaemia: prevalence and antimicrobial susceptibility patterns of common pathogens at Princess Marina Hospital, Botswana. Southern African Journal of Infectious Diseases. 2015 Sep 18;30(3):108–13.
5. Viswanathan R, Singh AK, Ghosh C, Dasgupta S, Mukherjee S, Basu S. Profile of Neonatal Septicaemia at a District-level Sick Newborn Care Unit. Journal of Health, Population and Nutrition. 2012 Jul 17;30(1).
6. Gkentzi D, Kortsalioudaki C, Cailes BC, Zaoutis T, Kopsidas J, Tsolia M, et al. Epidemiology of infections and antimicrobial use in Greek Neonatal Units. Archives of Disease in Childhood - Fetal and Neonatal Edition. 2019 May;104(3):F293–7.
7. Thomas J, Govender N, McCarthy KM, Erasmus LK, Doyle TJ, Allam M, et al. Outbreak of Listeriosis in South Africa Associated with Processed Meat. New England Journal of Medicine. 2020 Feb 13;382(7):632–43.
8. Richard Spoor INC. Listera Class Action [Internet]. 2018. Available from: https://listeriaclassaction.co.za/ (accessed June 11, 2021).
